# Retrospective evaluation of cotrimoxazole use as preventive therapy in people living with HIV/AIDS in Boru Meda Hospital

**DOI:** 10.1186/2050-6511-15-4

**Published:** 2014-02-08

**Authors:** Berhanu Geresu, Desye Misganaw, Yeshiwork Beyene

**Affiliations:** 1 Department of Pharmacy, College of Medicine and Health Sciences, Wollo University, Dessie, Ethiopia; 2 Department of Nursing, College of Medicine and Health Sciences, Wollo University, Dessie, Ethiopia

**Keywords:** Drug use evaluation, Cotrimoxazole, Preventive therapy, HIV/AIDS, Boru Meda

## Abstract

**Background:**

Drug use evaluation is a performance improvement method that focuses on evaluating and improving drug use process to achieve optimal patient outcomes. Drug use evaluation helps in identifying, preventing or resolving actual and potential drug related problems. The objective of the study was to evaluate the use of cotrimoxazole as preventive therapy in people living with HIV/AIDS in Boru Meda Hospital, Northeast Ethiopia.

**Methods:**

A retrospective drug use evaluation was conducted on patients’ medical history records based on a validated drug use evaluation criteria according to the national guideline. Medical history records of 248 patients were selected using systematic sampling method.

**Results:**

The result showed that 49.6% of the patients were at WHO clinical stage III at the start of cotrimoxazole preventive therapy. In this study, the use of cotrimoxazole preventive therapy was consistent with the guideline in the rationale for indication (97.98%), dose (96.77%), and its use despite the presence of contraindications (91.93%). Problems regarding drug-drug interaction were identified in 49.59% of cases, and 20.97% of patients discontinued cotrimoxazole preventive therapy due to different reasons.

**Conclusions:**

In most patients cotrimoxazole preventive therapy was consistent with the national guideline regarding the rationale for indication, dose, discontinuation and its use in the presence of contraindications.

## Background

Rational drug use is concerned with promoting quality of care and cost- effective therapy, preventing unnecessary exposure to side effects, maximizing therapeutic benefits, and improving patient compliance [1]. Drug use evaluation (DUE) is one of the methods used in combating the development of bacterial resistant to anti-microbial agents and improving therapy [2].

Cotrimoxazole (CTX), a fixed dose combination of sulfamethoxazole and trimethoprim, is a broad spectrum antimicrobial agent that targets a range of aerobic gram positive and gram negative organisms, fungi and protozoa [3,4]. CTX is preferable for both primary and secondary prophylaxis of pneumocystis jirvoeic pneumonia (PCP) in adults and adolescents [5,6]. The World Health Organization (WHO) and the Joint United Nations Programme on HIV/AIDS (UNAIDS) have recently recommended the use of co-trimoxazole prophylaxis for HIV-infected adults in Africa with symptomatic HIV disease (stage II, III or IV of the WHO classification of HIV infection and disease) and for asymptomatic individuals who have a CD4 T-lymphocyte count of ≤500×10^6^/l or total lymphocyte count (TLC) equivalent [7].

The recommended dose for adult and Adolescents is two single strength tablets of CTX (tablets of 80 mg Trimethoprim and 400 mg Sulphamethoxazole) or one double strength tablet (1 tablet of 160 mg Trimethoprim and 800 mg sulfamethoxazole) daily or three times per week. In addition, children in the age range 6 months-5 years or (5-15 kg) take (200 mg/40 mg) CTX, 6 years-14 years or (15-30 kg) take (400 mg/80 mg) CTX and >14 years or >30 kg take (800/60 mg) CTX. Adverse conditions that lead to discontinuation of CTX are itching (with mucocutaneous lesion and or fever), significant rash, fever, Steven Johnson syndrome and sever anemia [4].

CTX preventive therapy (CPT) is a simple well tolerated and cost effective intervention which can extend and improve the quality of life for people living with HIV/AIDS (PLWHA) including those on antiretroviral therapy (ART). CPT is associated with a 25-46% reduction in mortality among individuals infected with HIV in sub-Saharan Africa even in areas of high bacterial resistance to the antibiotic [7,8].

Despite the proven clinical benefits of CTX and recommendation by WHO, its routine use in developing countries particularly in sub-Saharan Africa has remain limited [8]. CPT is effective at preventing a number of opportunistic infections (OIs) among HIV positive individuals initiating ART. Although the drug is cheap and widely available, many countries failed to implement policies to provide nation wide coverage of the drug [9]. Use of judiciously selected drugs with a valid set of guidelines will bring success not only to health care system of the country but also to the whole socio-economic development [6].

Antibiotics represent approximately 30% of the acute hospital care expenditure and they are prescribed for 20-50% of patients. The development of drug resistant organisms may emerge as a result of many factors including irrational use of drugs [2]. The inevitable consequences of the emergence of antimicrobial resistant pathogens fuels an ever increasing need for new drugs and contribute to the rising cost of medical care [10].

Retrospective DUE addresses issues of indications, contra indications, drug interactions, dosage, therapeutic duplications and patient monitoring patterns. In addition it provides information that opens ways to provide feedback for prescribers on their performance in implementing treatment protocols and their compliance with preset approved guidelines for the use of each medication. In dealing with the fight against health problems like HIV/AIDS, that have deep rooted socio-economic impact, it is quite useful to evaluate the effectiveness of programs for better outcomes. Thus DUE should be incorporated as one of such monitoring systems in the health care system of the country in response to the issue of AIDS epidemic and OPIs [11]. Therefore, the aim of this study was to evaluate the use of cotrimoxazole as preventive therapy in PLWHA Boru Meda Hospital.

## Methods

A retrospective drug use evaluation was conducted on patients’ medical history records in Boru Meda Hospital (BMH), Northeast Ethiopia from May 22-30/2012. The hospital is a rural hospital located 4 kilo meters away from Dessie town and comprised of 6 general practitioners, 40 nurses, 16 pharmacy technicians and 4 X-ray technicians with a total beds of 80. A total of 701 patients were receiving CPT before February 2012. Medical history records of 248 patients out 701 were selected using systematic sampling method from sequentially arranged medical records.

The independent variables were age, sex, patients’ clinical condition and laboratory results. Whereas the dependent variables include indication, contraindication, drug-drug interaction, dosage and discontinuation of CPT.

Data collection format containing the variables to be measured was developed and used to collect data. The collected data were filtered, categorized and the results were analyzed using SPSS version 15, interpreted and presented using tables and charts.

Ethical clearance was obtained from College of Medicine and Health Sciences, Wollo University Institutional Review Committee (IRC), and permission was sought from BMH.

## Results

Out of 248, 123 (49.60%) patients were at WHO clinical stage III HIV infection at the start of CPT. Even though co morbid illnesses were not documented for most (67%) of the patients, the prevalence of tuberculosis (TB) and fungal infections were 11.69% and 12.50%, respectively. Majority of the patients 221 (89.11%) were found to have CD4 count of less than 350cell/mm^3^ (Table 1).

**Table 1:** Baseline characteristics of PLWHA at the start of CPT on BMH before February 2012

**Conditions**	**Variables**	**Number (%)**
HIV infection clinical stage	I	37 (14.92)
	II	66 (26.61)
	III	123 (49.60)
	IV	22 (8.87)
Co morbid illness	TB	29 (11.69)
	Fungal infection	31 (12.75)
	PCP	2 (0.8)
	Others	18 (7.26)
Laboratory results	CD4 count <350 cell/mm^3^	221 (89.11)
	Hemoglobin <7 g/dl	4 (1.61)
	Platelet count < 50,000 cells/dl	4 (1.61)
	Neutrophil count < 750 cells/dl	3 (1.2)
	ALT > 115 IU/L (for males) >90 IU/L (for females)	3 (1.2)

ALT- alanine transaminase, IU- international unit.

The result also showed that 243 (97.98%) patients were in line with the national CPT guideline regarding to indication to start and 240 (96.77%) patients received correct dose of CTX (Table 2). The medical history cards showed that 20 (8.06%) patients were using CTX against contraindications out of which 5 (25%) developed sever anemia (Table 3).

**Table 2:** Cotrimoxazole dosage prescribed for PLWHA in BMH before February 2012

**Age distribution**	**Number (%) of cases**		
	**Appropriate dose, number (%)**	**Inappropriate dose, number (%)**	**Total**
<6 month	3 (1.2)	_	3 (1.2)
6 month-5 yr	4 (1.61)	4 (1.61)	8 (3.23)
6-14 yr	14 (5.64)	4 (1.61)	18 (7.25)
15-49 yr	202 (81.45)	_	202 (81.45)
>50 yr	17 (6.85)	_	17 (6.85)
**Total**	**240 (96.77)**	**8 (3.23)**	**248 (100)**

**Table 3:** Cotrimoxazole use despite the presence of contraindications among PLWHA in BMH before February 2012

**Condition evidence for contra use of CPT**	**Number (%)**
Severe anemia (Hg < 7 g/dl of blood)	5 (25)
Elevated liver enzymes (SGPT > 115 IU/L for males and 90 IU/L for female)	3 (15)
Severe neutrophil (<750 cell/dl)	3 (15)
Severe thrombocytopenia (<50,000 cells/dl)	4 (20)
1^st^ trimester of pregnancy	5 (25)
Sulfa allergy	_
**Total**	**20 (8.06)**

In 123 (49.6%) cases CTX interaction with zidovudine (AZT) was documented and there were no other drug-drug interactions involving CTX. Among 52 patients who discontinued CPT, 10 (19.25%) and 14 (26.92%) were due to improvement of CD4 (>350 cells/mm^3^) and peptic ulcer disease, respectively (Table 4).

**Table 4:** Common reasons for discontinuation of CPT among PLWHA in BMH before February 2012

**Reason for discontinuation**	**Number (%)**
Peptic ulcer	10 (19.21)
Skin rash	6 (11.53)
Sever anemia	3 (5.77)
Pregnancy	6 (11.53)
CD4 > 350 cells/mm^3^	14 (26.92)
Others (not recorded)	13 (25.00)
**Total**	**52 (20.97)**

## Discussion

One of the most pressing problems facing health providers and administers in many countries is insuring rational drug use, which implies an individual approach to patient treatment. The presence of standard treatment guidelines and drug formularies for selected drugs in a health facility does not ensure that drugs are prescribed and used correctly. One mechanism to ensure correct use of drug is DUE [2].

In this study the use of CTX among PLWHA was consistent with the national guideline in the rationale for indication in most (97.98%) patients. In 2% of the patients (who were WHO clinical stage I and CD4 level >350 cells/ml) CPT was started without any symptomatic disease. The CTX dosage prescribed for most patients were appropriate except in 1.6% of patients in the age range 6 months to 14 years. This might leads to insufficient and inappropriate treatment.

The finding on the rationale for indication and appropriate dose were in line with the study conducted in Jimma University Specialized Hospital in which all cases of the indication to start and dose were according to the national guideline [12]. On the contrary, the result for CTX dose was slightly different from the study done in Hawasa referral hospital in which 87% of the cases on usage of CTX dose were according to the national guideline [10].

CTX was used despite contra indications in cases of 8.06% and in 91.93% of cases the drug was used without any contraindications. This is in agreement with a research done in Jimma [12], which showed 98.3% of the cases were in accordance with the guideline. The national guideline suggests the importance of monitoring drug usage for patients with severe anemia taking CTX and AZT [1]. In 49.59% of cases CPT had interactions with AZT, which is far from 98.3% of patients in Jimma [12]. The interaction of these drugs causes bone marrow suppression and thereby hematological abnormalities [13]. This in turn results in lack of adherence and poor patient outcomes.

WHO and the Federal Ministry of Health of Ethiopia recommended the reasons for discontinuation of CPT in PLWHA for better treatment outcome and for preventing potential drug related problems [6,8]. Among 52 patients who discounted CPT, 25% of them were without any documented data for their discontinuation and 23% of them discontinued due to both skin rash and pregnancy. Most patients discontinued CPT due to peptic ulcer (19.25%) and CD4 improvement (26.92%). The result for discontinuation of CPT (75%) which was in agreement with the national guideline and with the study done in Jimma University Specialized Hospital [12].

Patients discontinue CPT when their CD4 count was restored to the recommended level and this in turn decreases pill burden in patients taking ART and thereby increases treatment adherence. If discontinuation is not according to the guideline, it may lead to antimicrobial resistance, fuelling an ever increasing need for new drugs and contributing the rising cost of medical care.

To date, there are no published studies conducted on evaluation of cotrimoxazole use for prophylaxis in PLWHA outside Ethiopia and hence it was not possible to compare the practice of this hospital with practices of other clinical settings. The findings were discussed in relation to the 2005 CPT guideline of Ethiopia and few studies done in Ethiopia.

## Conclusions

In summary the use of CPT among PLWHA was consistent with the national guideline regarding to rationale for indication, dose, discontinuation and its use despite the presence of contraindications in most patients, however, problems regarding drug- drug interaction were identified in more than half of the patients.

## Competing interests

The author(s) declare(s) that they have no conflicts of interest to disclose.

## Authors’ contribution

All have been involved in the design and conceptual framework of the study. BG worked on analyzing the data; DM was involved in drafting the manuscript, and the role of YB was crucial in data collection and manipulation. All the authors have read and approved the final manuscript.

## Authors’ information

BG is a pharmacologist working at Wollo University as instructor, researcher and head of department of pharmacy. DM and YB are a pharmacist and a nurse working at Wollo University as an instructor and a researcher, respectively.

## Pre-publication history

The pre-publication history for this paper can be accessed here:

http://www.biomedcentral.com/2050-6511/15/4/prepub
